# Stage-specific transposon activity in the life cycle of the fairy-ring mushroom *Marasmius oreades*

**DOI:** 10.1073/pnas.2208575119

**Published:** 2022-11-07

**Authors:** Markus Hiltunen, Sandra Lorena Ament-Velásquez, Martin Ryberg, Hanna Johannesson

**Affiliations:** ^a^Department of Organismal Biology, Uppsala University, SE-752 36 Uppsala, Sweden

**Keywords:** transposable elements, genome evolution, basidiomycete, life cycle, mutation accumulation

## Abstract

Genetic variability can be generated by different mechanisms, and across the life cycle. Many basidiomycete fungi have an extended somatic stage, during which each cell carries two genetically distinct haploid nuclei (dikaryosis), resulting from fusion of two compatible monokaryotic individuals. Recent findings have revealed remarkable genome stability at the nucleotide level during dikaryotic growth in these organisms, but whether this pattern extends to mutations affecting large genomic regions remains unknown. Furthermore, despite high genome integrity during dikaryosis, basidiomycete populations are not devoid of genetic diversity, begging the question of when this diversity is introduced. Here, we used a *Marasmius oreades* fairy ring to investigate the rise of large-scale variants during mono- and dikaryosis. By separating the two nuclear genotypes from four fruiting bodies and generating complete genome assemblies, we gained access to investigate genomic changes of any size. We found that during dikaryotic growth in nature the genome stayed intact, but after separating the nucleotypes into monokaryons, a considerable amount of structural variation started to accumulate, driven to large extent by transposons. Transposon insertions were also found in monokaryotic single-meiospore isolates. Hence, we show that genome integrity in basidiomycetes can be interrupted during monokaryosis, leading to genomic rearrangements and increased activity of transposable elements. We suggest that genetic diversification is disproportionate between life cycle stages in mushroom-forming fungi, so that the short-lived monokaryotic growth stage is more prone to genetic changes than the dikaryotic stage.

## Significance

The life cycle in many forms of multicellular life has an extended period of somatic growth between sexual generations, which may allow many genetic variants to accumulate. The longer the somatic growth phase is, the more variation is expected to arise during this phase. Despite this, long-lived fungi have been shown to accumulate very little variation during somatic growth. The life cycle of these fungi also includes a brief somatic stage after meiosis, called monokaryosis. Here, we show a burst of proliferation of selfish genetic elements during monokaryosis in the fairy-ring mushroom, *Marasmius oreades*. We suggest that the generation of genetic diversity between life cycle stages may be imbalanced, with some stages more prone to genetic changes than others.

**Fig. 1.**
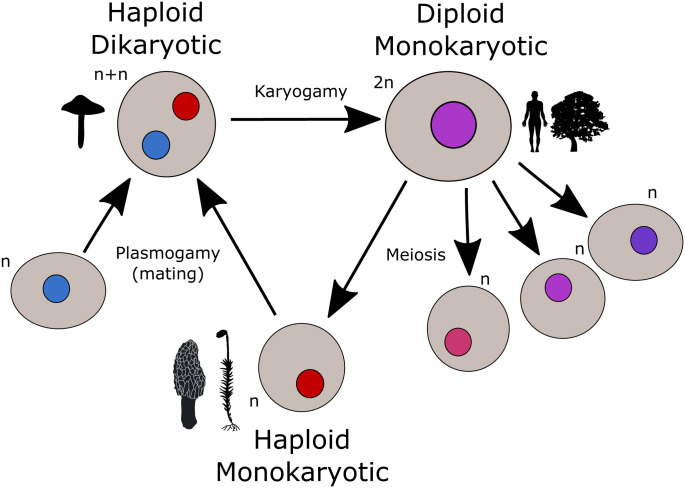
Generalized eukaryotic life cycle. The majority of sexual eukaryotes cycle between haploid and diploid stages throughout their life cycle. Most animals and vascular plants spend the majority of their life as diploid, while in bryophytes, many single-celled eukaroytes, and many fungi, the haploid stage dominates the life cycle. The dikaryotic stage is extremely short-lived in most organisms, where plasmogamy immediately follows by karyogamy and, hence, a single nuclear type is present in each cell (i.e., monokaryosis). However, many fungi, in particular mushroom-forming basidiomycetes, show an extended time between plasmogamy and karyogamy, leading to an extended dikaryotic stage where two haploid nuclei coexist within cells.

Genetic variability is a prerequisite for evolution. It can be generated during the entire life cycle, and how new variation is accumulated and maintained will depend on factors, such as the time and number of cell divisions taking place at each life cycle stage, and on the pattern of inheritance. Most forms of multicellular life employ mixed reproductive strategies, often including extended periods of clonal growth and propagation between occasional sexual reproductive events (1). Thus, somatic genetic variation (defined here as variation generated over growth and development) can have great evolutionary impact, both in terms of individual fitness and inherited variation. Theory predicts that the longer the somatic growth phase is, the more important it will be for accumulating variation (2–6). However, recent studies have indicated that despite having somatic growth stages extending up to thousands of years, some plants and fungi accumulate very little genetic variation during this part of the life cycle (7–11), which is also consistent with the negative correlation between somatic mutation rate and longevity in mammals (12). Life cycle characteristics vary enormously across the tree of life (Fig. 1), and the relative contribution of different life cycle stages to new genetic variation remains poorly understood and needs to be explored further.

The processes acting as generators of variation can be many, with the most obvious ones being mutation and meiotic recombination. In addition to canonical mutation and sexual recombination, some organism groups diversify their genetic material through asexual processes, such as mitotic recombination and parasexuality (13–19). An important type of mutation is the movement of transposable elements (TEs; transposons), which are selfish genetic elements capable of independently proliferating in their host genome. These are well known as drivers of genomic size and diversity in a multitude of different organism groups (20–27), in both somatic and sexual tissues (23, 27–30). Recently, the study of spontaneous mutation accumulation has exploded in a plethora of different organisms, especially with a focus on single-nucleotide variants (7–9, 31–35). Large-scale genomic changes, such as structural variants (SVs), TE insertions, and mitotic processes of recombination, are known to be of great importance for adaptive evolution (36–40), but the timing of such events during the life cycle is less well explored.

**Fig. 2.**
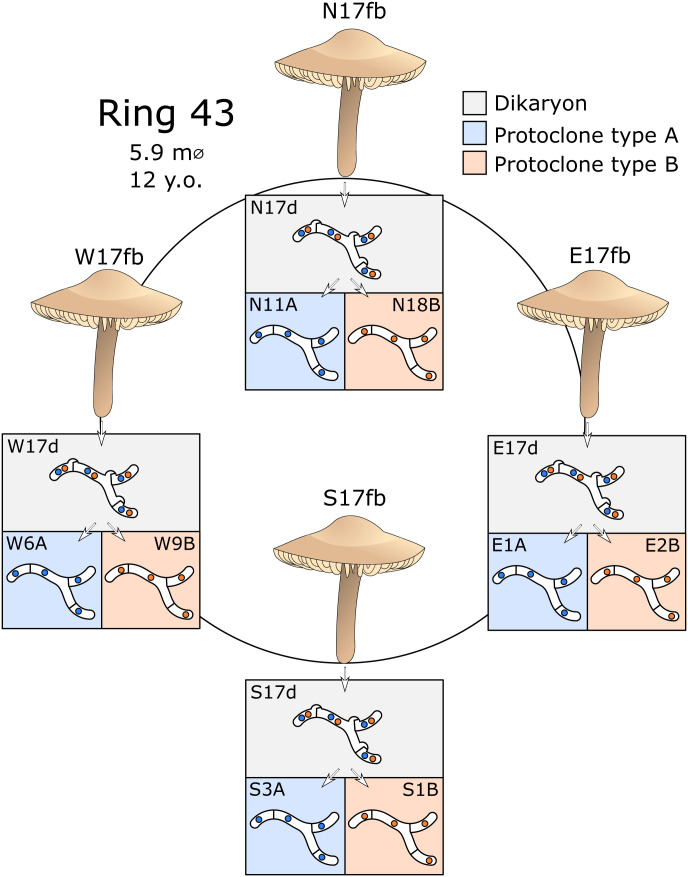
Sampling strategy and strain isolation. Four fruiting bodies (fb) were collected from the cardinal directions of a *M. oreades* fairy ring: north (N), east (E), south (S), and west (W). Pure cultures for which dikaryosis (d) was verified by clamp connections at hyphal septa, were isolated from stipe tissue of the fruiting bodies. From these four dikaryotic cultures, protoplast isolation and regeneration was used to recover eight monokaryotic isolates (protoclones, numbered after laboratory isolation) of both constituent nuclear genotypes (nucleotypes, here A or B, defined by allele sequence at the homeodomain mating type locus).

In this study, we investigated generators of variation at different life cycle stages of the fungus *Marasmius oreades*. This species is a basidiomycete fungus with a sexual cycle where haploid progeny in the form of basidiospores are generated. The basidiospores germinate to form monokaryotic, haploid mycelia, which are likely to be transient in nature. After mating (plasmogamy) between two monokaryons of compatible mating type, the resulting mycelium enters an extended dikaryotic phase, carrying two genetically distinct haploid nuclei per cell, before going through nuclear fusion (karyogamy) and meiosis in the fruiting bodies (mushrooms), where basidiospores are produced (41, 42). Growth of *M. oreades* occurs in characteristic “fairy rings,” which are circular, underground mycelia extending outward over time. Each fairy ring has a common origin at the mating event and can thus be considered a genetic individual (42, 43). Different sectors of the ring differ only by the mitotic mutations, recombination, or other genomic changes that have occurred during growth of the ring (8), and comparisons between different parts of the ring can reveal such changes. We utilized a hierarchical sampling strategy, from the haploid monokaryon up to population level, and focused on large-scale changes to the genome (i.e., transpositions, SVs, and signatures of mitotic recombination). We sampled fruiting bodies from one fairy ring of *M. oreades* at four different locations, isolated dikaryotic mycelia from the fruiting bodies into pure culture, and separated their haploid nuclei through nonmeiotic protoplast regeneration into monokaryotic isolates (Fig. 2) (44–47). By utilizing long-read Nanopore PromethION sequencing (48), we were able to fully reconstruct the whole genome sequences of these monokaryons and their source dikaryons. The dataset was supplemented with our previously generated whole-genome sequencing data of a local *M. oreades* population (8), in addition to 95 single-spore isolates (49), allowing us to study new genetic variation at each level, from individual nucleus up to population.

Our results confirmed previous findings of an extremely high genomic stability during dikaryotic growth of *M. oreades* fairy rings (8): no new variants of 50 bp or larger could be inferred to have arisen during dikaryosis, and no mitotic cross-over or exchange between nuclei had taken place, despite the fairy ring under study being around 12 y of age at sampling. However, we found considerable amounts of new genetic variation in monokaryotic isolates, originating either from protoplasts or from basidiospores. These variants were in the form of TE insertions and excisions, in addition to SVs of up to several hundred kilobases in size. Based on these findings, we suggest that genetic variation in mushroom-forming fungi is mainly generated at the monokaryotic, haploid stage of the life cycle, and that once formed, the dikaryon is remarkably resistant to any type of genotypic change. Thus, contrary to expectations, the importance of the different life cycle stages can be highly disproportionate for introducing new variation, highlighting the importance of investigating the entire life cycle for understanding how genetic variation is generated and maintained in eukaryotes.

## Results

We sampled and analyzed data from four fruiting bodies of an *M. oreades* fairy ring located in Uppsala, Sweden (designated ring 43, Ø 6 m, ∼12 y old). From these four fruiting body collections, obtained from the cardinal directions of the ring, we isolated dikaryotic cultures from stipe tissue and used protoplast isolation and regeneration to separate the monokaryotic strains (protoclones) carrying either one of the two nuclear genotypes (nucleotypes) of the dikaryon (44) (Fig. 2 and *SI Appendix*, Table S1). The nucleotype of each protoclone was defined based on allelic identity (A or B) at the homeodomain mating type (HD) locus (e.g., N11A) (*SI Appendix*, Table S1). From each fruiting body, protoclones of both nucleotypes were isolated, resulting in a total of eight monokaryotic strains in addition to their four dikaryotic source strains of ring 43 (Fig. 2 and *SI Appendix*, Table S1). We used Illumina NovaSeq to generate genomic data from the tissue of the fruiting bodies, and both Illumina NovaSeq and Nanopore PromethION sequencing to obtain whole-genome sequences of the isolated strains (*SI Appendix*, Table S1). *k*-mer spectra of the raw Illumina data supported the expected nuclear state (mono- or dikaryosis) of the strains (*SI Appendix*, Fig. S1).

### No Genetic Exchange between Constituent Nuclei during Dikaryotic Growth.

**Fig. 3.**
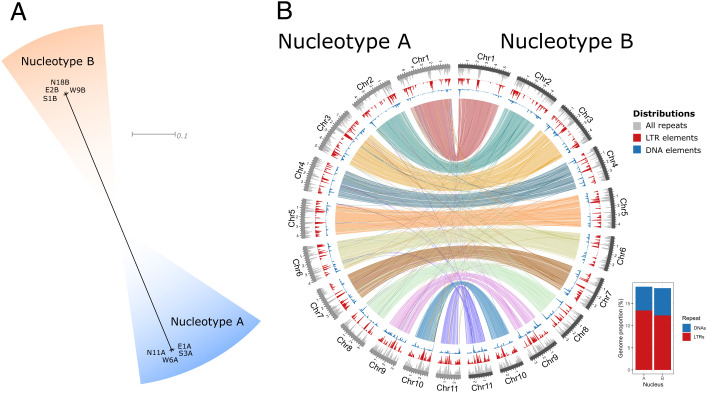
Genome comparison between nuclear types within the fairy ring under study. (*A*) Phylogenetic network of the protoclones, constructed from genome-wide variants, showing a clear separation of nucleotypes compared to variation within the same nucleotype. Branches are drawn proportional to the scale bar (substitutions per site). (*B*) Whole-genome alignment between combined assemblies of the nucleotypes. Alignments are shown as links colored by chromosome. Alignments smaller than 20 kb were removed for clarity. Distributions calculated in windows of 50 kb and steps of 10 kb for all repeats, LTR, and DNA elements are shown as internal tracks. (*Inset*) Percentage of DNA- and LTR elements in the two genomes.

We started out by investigating the relationships between the monokaryotic protoclones from the different parts of the ring, using variants identified in the Illumina data. They formed two distinct groups corresponding to the two nucleotypes, with no evidence of mitotic recombination, gene conversion, or other genetic exchange between the nuclei of the dikaryon over vegetative growth of the ring (Fig. 3*A*). Next, we generated combined genome assemblies, where we utilized Nanopore and Illumina sequencing reads from all protoclones of each nucleotype; these samples should only differ by mutations that had arisen during growth of the fairy ring or during protoplast regeneration. Assembling the two pools of reads resulted in gapless, near-complete assemblies of the expected 11 chromosomes with high quality at the base level (*SI Appendix*, Table S2). These two “consensus” genome assemblies were assumed to represent the ancestral nucleotypes A and B of the fairy ring, and were used downstream as references for analyses of individual protoclones. With these data in hand, we investigated the level of structural difference between the nucleotypes A and B using the combined assemblies, and found that they were largely collinear at the macrolevel (Fig. 3*B*). The repeat content of both genotypes was dominated by long terminal repeat (LTR) elements, and to a lesser degree by DNA transposons, as observed previously in this species (49). The nucleotide diversity (π) in the fairy ring under study was 0.0056, similar to the population average of 0.0057 [based on data from Hiltunen et al. (8)], indicating that the nucleotypes in ring 43 were not more distantly related to each other than expected by random mating in the population.

### Stark Contrast in Transposition between Mono- and Dikaryons.

In order to investigate transposon activity in our sample, we generated individual genome assemblies of all monokaryotic protoclones, resulting in assemblies of comparable quality to the combined assemblies (*SI Appendix*, Table S2). Through multiple approaches for variant detection in the individual genome assemblies and raw Nanopore reads (*SI Appendix*, Fig. S2), we were able to identify a total of 72 different insertions, deletions, or SVs, using 50 bp as a threshold for minimum variant size (Dataset S1). Most of the new variants were caused by TE insertions. Notably, we recovered the 72 variants only in the protoclones, and no variants were found in the dikaryotic strains or fruiting bodies. This finding revealed that although the age of the *M. oreades* genet under study was estimated to be ∼12 y, providing ample opportunity for mutation, no SV could be inferred to have arisen during growth in nature. Instead, the variants were introduced during or after protoplast isolation to spread in the regenerated mycelia prior to DNA extraction. Moreover, most of the discovered variants were found in a subset of the Nanopore reads from a single protoclone strain, indicating multiple genotypes in the sample. Having verified that the protoclones were monokaryotic, it is likely that these variants were derived after the single-cell bottleneck at the protoplast to form a polymorphism in the cell population during propagation of the mycelium before sequencing, making the mycelium as a whole a mosaic of genotypes at those loci. Based on this finding, we grouped the 72 variants into the categories “fixed” (fewer than three reads with the reference allele; *n* = 12) (*SI Appendix*, Fig. S3*A*) or “mosaic” (*n* = 60) (*SI Appendix*, Fig. S3*B*). Most of the mosaic variants escaped assembly and were only identified by mapping back reads to the combined assemblies of the nucleotypes.

The finding that new TE insertions and other SVs were introduced only in the monokaryotic protoclones led us to suspect that the stressful events of protoplast isolation and regeneration caused genome instability, resulting in new insertions and deletions (50). If so, new TE insertions should be a lot more common in protoplast-derived monokaryons than in monokaryons derived through the “natural” means of meiosis and basidiospore generation. To investigate this question, we utilized our previously generated whole-genome dataset of 95 single-basidiospore isolates (49). These samples originated from the same fairy ring as the protoclones but were collected from nature 1 y after the protoclone strains. Despite the dataset being composed of short-read Illumina sequences with a lower TE insertion discovery rate than long reads, we found 37 insertions in the single-spore–derived monokaryons. This finding revealed that the method of protoplast isolation and regeneration on its own is insufficient as an explanation for the increased activity of TEs in the protoclones.

### Characterization of Transposon Insertions Revealed Active *hAT*, Mariner, and Long Interspersed Nuclear Element (LINE) Families.

**Fig. 4.**
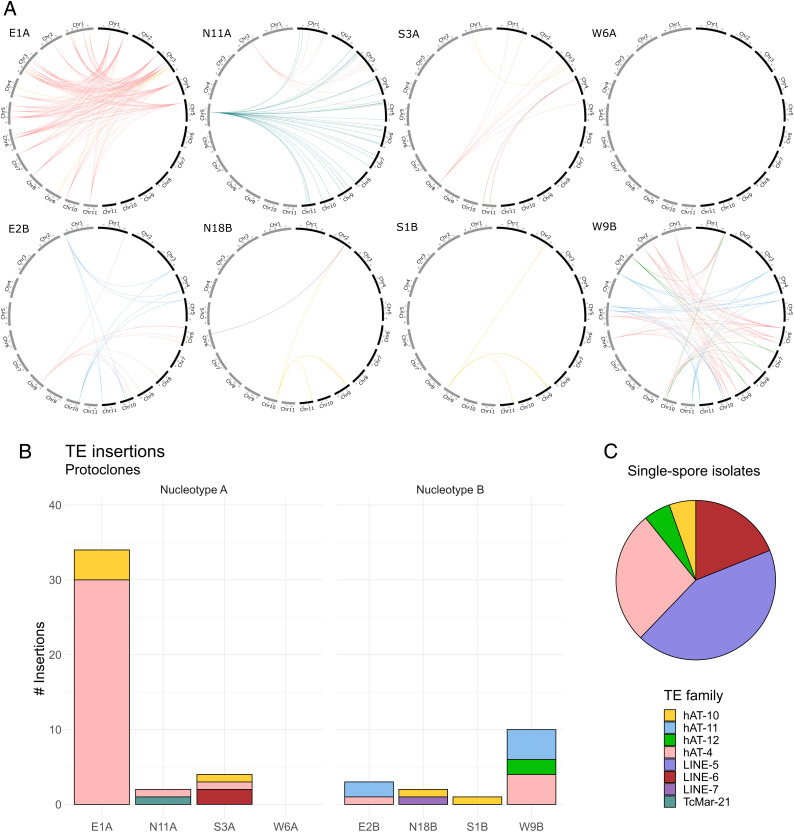
Novel transposon insertions. (*A*) Genomic locations of new insertions and original copies. In each panel, the left chromosomes (in gray) depict the protoclone while the right chromosomes (in black) show the respective combined assembly. (*Upper*) Protoclones of nucelotype A; (*Lower*) protoclones of nucleotype B. Links connect new transposon insertions on protoclone chromosomes (*Left*) to the positions of these elements in the combined assembly of the putatively ancestral genotype (*Right*). For example, in N11A, there was a single insertion of MarorTcMar-21 on chromosome 5, with 32 potential source copies in the ancestral genome (these copies are also still present in N11A but not drawn), and in E1A, there were two new insertions of MarorhAT-10 on chromosomes 4 and 9, but only a single copy in the ancestral genome (in yellow), meaning that this copy has to be the source of the new insertions. Note that links do not depict alignments and are not drawn to scale. (*B*) Absolute numbers of new insertions in the protoclones. (*C*) Distribution of new insertions in single-spore isolates. Color coding is common for all subplots.

**Fig. 5.**
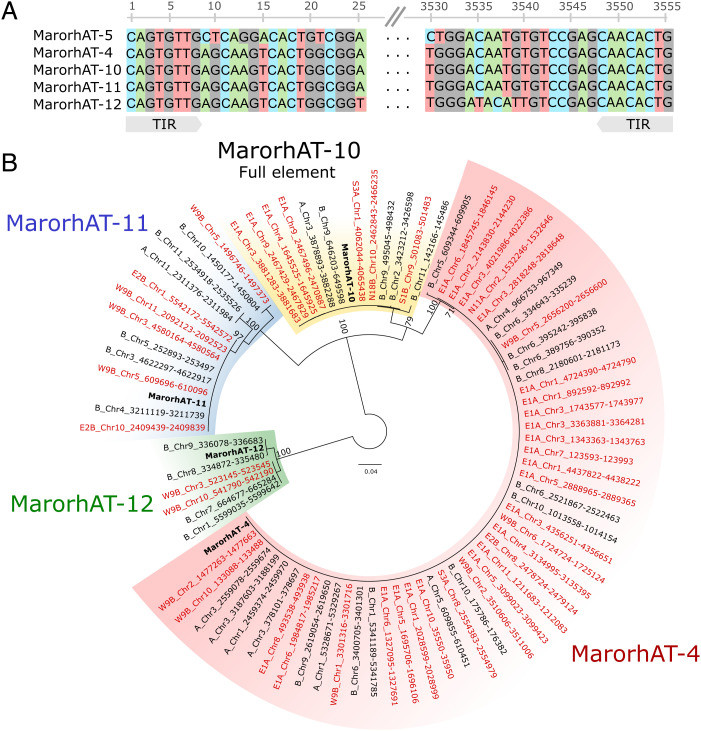
Identification of active *hAT* type elements. (*A*) Alignment of the TIRs of consensus sequences of similar *hAT* type elements in the protoclone genomes. (*B*) Maximum-likelihood genealogy of MarorhAT-10 and related nonautonomous elements. Family consensus sequences are highlighted in bold. New insertions not found in the original genotype are highlighted in red. The genealogy was arbitrarily rooted with MarorhAT-12. Branches are drawn proportional to the scale bar (substitutions per site), with bootstrap support values shown above (tip values are ignored for clarity).

Of the 72 variants detected in the monokaryotic protoclones (Dataset S1), at least 63 were directly related to transposon insertions (*n* = 49), deletions (*n* = 13), or inversions (*n* = 1). The remaining nine did not appear to be caused by TE activity, but included translocations of hundreds of kilobases in E1A and S1B in regions rich in repeats near the ends of chromosomes, as well as additional large (12 to 18 kb) and small (70 to 300 bp) deletions. Of the transposon-related variants, the highest number was detected in the strain E1A, but all protoclones except W6A had evidence of active transposition (Fig. 4). Many TE variants had sequence similarity to previously characterized *hAT* element families (49) (Fig. 5). We identified insertions and excisions belonging to the known nonautonomous MarorhAT-4 family, both in fixed and mosaic state. In addition, we found three new families that were similar in sequence to MarorhAT-4 and named them MarorhAT-10, MarorhAT-11, and MarorhAT-12. The new families had close resemblance to MarorhAT-4 in the terminal inverted repeats (TIRs) at their 5′ and 3′ ends (Fig. 5*A*). The family MarorhAT-10 (3,396-bp long) carried a long internal section that was absent in the other hAT families (597 to 631 bp). Within this section we identified a *hAT* type transposase domain (l05324), indicating that this element is capable of autonomous transposition, which was lacking in the families without the internal section. By extracting all complete copies of these *hAT* families from the protoclones and combined assemblies and making a phylogeny (Fig. 5*B*), we were able to pinpoint the original copies that gave rise to the new insertions of MarorhAT-10 and MarorhAT-11. In the case of MarorhAT-10, there was only a single copy in the original A nucleotype at chromosome 3. This copy had given rise to new insertions in E1A chromosome 9 and S3A chromosome 1. The original site of MarorhAT-10 in chromosome 3, in addition to all copies of MarorhAT-4, is mosaic in E1A, as expected if the element has excised in some cells, further showcasing its active transposition. In the original B nucleotype, MarorhAT-10 had four copies, and our phylogeny indicated that the new MarorhAT-10 insertions in N18B and S1B originated from either of the two copies on chromosome 9. For MarorhAT-11, we found one new insertion in W9B, which had 100% sequence similarity to another copy on chromosome 10, suggesting that this copy was the source for the new insertion (Fig. 5*B*).

The remaining TE insertions were similar to the previously characterized MarorLINE-6 [of the *Inkcap* superfamily (51)], MarorLINE-7 (*Tad1*), and MarorTcMar-18 (*Mariner*) families (49). The two MarorLINE-6 insertions were found in S3A, and were of different length at the 5′ end. We did not find any protein domains in the sequences of these insertions, but the sequence is similar to MarorLINE-5, which does contain a non-LTR–like reverse-transcriptase domain (cd01650). In the combined assembly of the A nucleotype, MarorLINE-6 has only a single copy, which must have acted as master copy for the new insertions in S3A. Similarly, MarorLINE-7, which had a new insertion in N18B, had a single copy in the original B nucleotype. This element encodes the reverse transcriptase itself, hence is probably moving autonomously. Finally, the new insertion of the *Mariner*-type element was observed in N11A, bore similarity to the MarorTcMar-18 family, but was shorter (1,120 compared to 3,972 bp) and lacking protein domains. Many copies of this element were found in the A nucleotype combined assembly (Fig. 4*A*) (N11A). We drew the conclusion that this element is a nonautonomous derivative of the MarorTcMar-18 element, and defined a new family called MarorTcMar-21. Notably, activity of mostly the same TE families as in the protoclones was detected the single-spore isolates (Fig. 4*C*). Specifically, in these strains, the insertions were caused by the MarorhAT-10 (*n* = 2), MarorhAT-4 (*n* = 10), MarorhAT-12 (*n* = 2), MarorLINE-5 (*n* = 16), and MarorLINE-6 (*n* = 7) families.

The sequence at five of the deletion events was related to either of the previously described TE families MarorPlavaka-20, MarorGypsy-26, MarorHelitron-2, MarorIS3EU-2, or MarorCMC-10 (49). These deletions do not appear to correspond to excised elements. Instead, in the case of the *Plavaka* element, the deletion does not encompass the whole 3′ end of the TE sequence, but includes additional sequence at the 5′ flank. As for the MarorGypsy-26 deletion in E1A, only one of the LTR sequences remains. LTR elements are known to occasionally delete one LTR and the inner sequence through nonallelic homologous recombination between the LTR sequences (52), which appears to have happened in this case. In the remaining cases, there were two copies of the elements in head-to-head orientation in the combined assembly. The deletions correspond roughly to one whole copy of the two, and are likely caused by nonallelic homologous recombination between the copies, resulting in the deletion of one of the copies and the sequences between them. The inversion happened in S1B in a region where the other protoclones carried two copies of the element MarorhAT-10 in head-to-tail orientation, separated by ∼2.5 kb of unrelated sequence. In S1B, one of these copies is inverted, and the middle sequence deleted.

### The Active TEs Contribute to Genetic Diversity at the Population Level.

We investigated the contribution of the identified actively transposing TE families to genetic diversity at the population level of *M. oreades*. Reasoning that if the TEs are active in the population, there should be a high variance in load between different genomes in the population. We investigated the fairy ring under study in addition to six other Swedish *M. oreades* rings for which the genomes were published previously (8). We quantified TE load in each genome by mapping Illumina reads (normalized to 35× coverage by random subsampling of reads) to the *M. oreades* repeat library (49) (with the additions of our newly discovered families) and measuring depth of coverage as a proxy for copy number of each TE family. We found that the coefficient of variation (CV; SD/mean) in depth of coverage varied to a high extent between different TE families (*SI Appendix*, Fig. S4). The variation in load of the TE families active in our data were generally high, with the CV of these families falling above the third quartile, with the exception of MarorhAT-4 and MarorTcMar-21. This result supported an active contribution to population diversity of the TEs that were active in our study, and also revealed a number of other families with high CV, indicating that these families may be active in other individuals in the population.

### Active Transposons Have Low Levels of Cytosine Methylation in Both Di- and Monokaryons.

Transposon activity is often negatively correlated with cytosine methylation at CpG sites (mCpG), and in fungi, suppression of TE movement is thought to be the primary function of cytosine methylation (53). Our Nanopore dataset allowed us to quantify mCpG frequencies in the dikaryons and their constituent monokaryotic protoclones (54, 55). As expected, we found repeat regions in both dikaryons and protoclones to be methylated to a much higher frequency than the rest of the genome, supporting that cytosine methylation may function to suppress TEs in *M. oreades* (*SI Appendix*, Fig. S5*A*). Averaged across the whole genome, repeat regions were slightly but statistically significantly less methylated in the dikaryons than in the protoclones (on average 0.75 and 0.76, respectively, of the reads per CpG site were methylated; *P* < 2.2e-16; *n* = 2,689,589 total sites; Wilcoxon rank-sum test). At the original copies of the MarorhAT-10 family, which we hypothesize drove the transposition of all *hAT* elements, mCpG frequencies were in some cases significantly different between the dikaryon and the constituent protoclone (*SI Appendix*, Fig. S5*B*). However, the dikaryons and protoclones were similar in having either high (i.e., close to 1) or much lower average mCpG frequencies at homologous TE sites (*SI Appendix*, Fig. S5*B*). The low-methylated copies had the highest sequence similarity to the newly inserted copies of MarorhAT-10 (Fig. 5), supporting that these copies acted as the sources for the new insertions. For the remaining TE families that were active in our study, mCpG frequencies varied to a much higher extent between different copies within the same genome than between homologous copies in different genomes, both for autonomous and nonautonomous families (*SI Appendix*, Fig. S5*C*).

## Discussion

In this study we investigated genomic rearrangements and TE mobilization in the fairy-ring mushroom *M. oreades* after 12 y of growth as a dikaryon in nature, after isolation to pure laboratory dikaryotic culture, and after separation of nuclear genotypes into monokaryons through protoplast isolation and regeneration. We found that during dikaryotic growth in nature, no mitotic cross-over, large-scale rearrangements, or transposition events had taken place in the *M. oreades* fairy ring under study. This result is in accordance with previous findings of an extreme genome integrity at the nucleotide level in *M. oreades* (8). We also did not find any large-scale genomic changes that had occurred during culturing of the dikaryotic mycelia in our study. In stark contrast, after separation of the nuclei into monokaryotic cultures (i.e., protoclones), several transposition events along with other structural variants could be identified, suggesting stage-specific differences in mutability. Most new TE insertions occurred in the protoclone E1A, driven primarily by MarorhAT-4 and MarorhAT-10, while W6A had no new insertions that we could find. This finding suggests that TE proliferation may occur in a burst-like manner, where activation of a single family (in this case MarorhAT-10) may lead to exponential increase of insertions of this and related nonautonomous elements. Similar bursts of transposition are known to have occurred during evolution in other lineages (e.g., salmonids) (56).

A potential explanation for the finding that transposition only occurred in monokaryons is that the stress imposed by digestion and regeneration of the cell wall during protoplast isolation had induced transposition. During protoplast regeneration, a medium with high osmotic potential is used, which may introduce additional stress. Previous research has shown that stress can promote the rise of structural variation, including transposition (57–60). In plants, protoplast isolation in particular is known for its mutagenic effect on TEs (61). In our study, however, the majority of the novel variants in the protoclones were present in a subset of reads per sample, showing that the sample consisted of mosaic genotypes. This result indicates that these variants arose at the growth stage after the single-cell bottleneck at protoplast isolation. We also identified transposition in single-basidiospore isolates. Note that the number of insertions that we found in these monokaryons is not directly comparable to the number in the protoclones, since they spent different amounts of time in culture during propagation, in addition to being sequenced by different technologies. Nevertheless, the finding that there had been active transposition also in spore-derived monokaryons showed that protoplast-induced stress was not sufficient to explain all transposition events in our study. Instead, our data suggest that the genome of *M. oreades* is unstable during monokaryosis per se, allowing genomic changes to accumulate during longer periods of growth as a monokaryon. It can be hypothesized that, as the monokaryotic condition is expected to be short in nature (e.g., ref. 62), selection for genome integrity primarily affects the dikaryotic life cycle stage, leading to monokaryons potentially being more susceptible to genomic rearrangements and other mutations. The mutational rate and spectrum are known to differ between haploid and diploid yeast (63), and our data suggest that the rate of mutations, including TE insertions, may differ between life stages with different nuclear compositions in filamentous fungi as well. Future experiments directly comparing monokaryons, derived from both protoplasts and basidiospores to dikaryons, in a controlled setting where cell divisions are tracked, should illuminate how much the rate of TE insertions differs between mono- and dikaryons of filamentous fungi.

One possible mechanism behind the difference in TE activity between mono- and dikaryons is a difference in the epigenetic landscape between the two nuclear conditions. Cytosine methylation is known to silence transcription of genes and transposons (64), leading to lower TE activity (65). One may hypothesize that in our *M. oreades* monokaryons, a relaxation in mCpG frequency had facilitated higher levels of transcription at transposon genes, subsequently leading to new TE insertions. In our dataset, however, we did not notice a systematic decline in mCpG frequency in monokaryons relative to dikaryons at the active transposon sites (*SI Appendix*, Fig. S5). Instead, mCpG levels at repeat regions were slightly higher in the protoclones. At the source, copies of the active TEs, low mCpG frequency was found when compared to other repeats in the genome, independent of nuclear condition (mono/dikaryon), and including most of the nonautonomous elements (*SI Appendix*, Fig. S5). These data support a correlation between methylation and TE activity, but do not explain the observed stage-specific activity. It is possible that some other epigenetic mechanism, such as modification of heterochromatin, was responsible for the difference in TE activity between mono- and dikaryons. Changes in the chromatin landscape during generation of the monokaryons may have activated TEs in those strains.

Genomic abundance of TEs is known to vary within fungal populations (22). Likewise, we found variation in TE load of many families in the *M. oreades* population, including most of the families that mobilized in our experiment (*SI Appendix*, Fig. S4). This finding indicates that these TE families may also be actively transposing in nature. The highest variation in load was found in *Helitron*, *Dileera*, and LINE families, and we hypothesize that these families may be active in other individuals in the population than the one we focused on in this study. A history of TE activity was also indicated by occasional disruption of chromosome synteny between the combined assemblies of nucleotypes A and B, coinciding with TE islands (Fig. 3*B*). Such a pattern may arise if these regions are affected by different TE insertion events in the population, causing them over time to diverge, especially if recombination is reduced in TE islands compared to the genome average (66).

Transposons can be autonomous or nonautonomous depending on their capability of proliferating on their own (67, 68). In our study, we observed the activity of the autonomous MarorhAT-10 and nonautonomous MarorhAT-4, MarorhAT-11, and MarorhAT-12 DNA element families. Nonautonomous DNA elements do not encode the transposase that is necessary for transposition themselves, instead parasitizing autonomous related elements by using their expressed transposase for proliferation (67). To be able to use the same transposase, the nonautonomous elements need to resemble their autonomous relatives in the TIR sequence to be correctly identified for excision (68). Accordingly, we found that the TIR sequences of these families were closely matching (Fig. 5*A*), and concluded that MarorhAT-4, MarorhAT-11, and MarorhAT-12 elements are derived from MarorhAT-10 and used the transposase expressed from it during our experiment. Similarly, the insertions of the MarorLINE-6 family were not predicted to encode any reverse transcriptase, and it is possible that this family uses the reverse transcriptase from the related family MarorLINE-5, which we found many new insertions of in the single-spore isolates. Retrotransposons of the LINE type are known to sometimes have incomplete transposition, resulting in truncation of the 5′ end (69). Accordingly, the insertions of the LINEs in our study were variable in length at the 5′ end. A similar result was recently found in the pathogenic fungus *Zymoseptoria tritici*, where most insertions were from truncated LINE retrotransposons (65). In addition to transposition, we identified large SVs in putative subtelomeric regions near the ends of chromosomes. Such regions are known to be especially vulnerable to ectopic recombination and rearrangements (70–72), with which our results agree.

In conclusion, we have shown that genome integrity is maintained at a structural level during dikaryotic, somatic growth of *M. oreades* fairy rings. Furthermore, genome integrity can be interrupted in monokaryons originating from both protoplasts and single spores, allowing for transposition and structural rearrangements to occur. Our results suggest that the generation of genetic diversity is disproportionate between mono- and dikaryotic life cycle stages, and may reconcile previous results of low mutation accumulation during dikaryotic growth, thus explaining patterns of genetic diversity in natural populations of fungi.

## Materials and Methods

### Sampling, Strain Isolation, and Culturing Conditions.

The sampling strategy of this study is schematically illustrated in Fig. 2. All strains originated from a single *M. oreades* fairy ring (ring 43) located in Berthåga, Uppsala, Sweden (59°51′27.3′′N, 17°34′19.2′′E). Four fruiting bodies were collected from July to September 2017, following the cardinal directions (i.e., one each from north, east, south and west; designated N17fb, E17fb, S17fb, and W17fb, respectively). The dikaryotic mycelia of the fruiting bodies were isolated to pure culture the following way. Stipes of the fruiting bodies were separated from the caps, surface sterilized in 70% ethanol for 10 to 20 s, dissected, and parts of them were placed on Petri dishes containing potato dextrose agar (PDA) and incubated at 25 °C. After 2 to 3 d, the stipe fragments were removed, and hyphae that had grown from the fragments into the agar were transferred to new PDA plates. We verified the nuclear condition of the dikaryons by both inspection for presence of clamp connections, which is a structure on the hyphae specific to dikaryotic growth, under the light microscope, and *k*-mer spectra of the raw sequencing data (see below). We refer to these isolates as dikaryotic cultures (strains N17d, E17d, S17d, W17d), containing nuclei of both genotypes, and they were maintained on PDA plates at 25 °C by subculturing approximately every 5 mo.

### Separation of Constituent Monokaryotic Strains through Protoplast Isolation and Regeneration.

From the four dikaryotic cultures, monokaryotic isolates of the two constituent nuclear types were obtained by protoplast isolation and regeneration. Dikaryotic cultures were grown in 2% liquid malt extract for 7 to 14 d before harvest. Mycelium was washed in sterile H_2_O and 0.5 M sorbitol. The cell wall degradation solution consisted of lysing enzymes from *Trichoderma harzianum* (Sigma; L1412) in 0.5 M sorbitol to a concentration of 5 mg/mL, and was sterile-filtered through 0.2-µm filters prior to use. The enzyme solution was added until the mycelium was completely covered, approximately 10 to 15 mL of solution. Degradation took place for 90 min at 30 °C, after which the solution was filtered through two layers of sterile Miracloth (Merck; 475855) into a sterile 2-mL microtube. The solution was kept on ice to reduce remaining enzyme activity. To wash off the enzyme, protoplasts were pelleted by centrifugation at 3,100 × *g* for 15 min at 8 °C, supernatant poured off, and tubes were filled up again with sterile 0.5 M sorbitol. The pellet was gently redissolved by flicking the tube and then pelleted again the same way. Supernatant was again poured off, but this time ∼100 µL was kept and spread onto a plate with regeneration medium (73), and incubated at 25 °C for up to a week. Plates were inspected daily for regenerated mycelia, which were transferred to new plates with PDA. Cultures were checked for clamp connections under a light microscope, and mycelia lacking clamps were genotyped at the HD mating type locus by PCR with allele-specific primers identified from previously published data (49) (98 °C 30 s initially, followed by 30 cycles of 98 °C 10 s, 60 °C 30 s, 72 °C 30 s, and final extension at 72 °C for 10 min; Phusion DNA polymerase; forward primer, common for both alleles: 5′-GACCATTTCTTTGCGTGAGC-3′; reverse primers: 5′-TTCATCTCCGCCTGTAGCTT-3′ and 5′-TGTGACGCTGCTTACCTACG-3′). In order to confirm that our method was successful in isolating single mating-type protoclones, the protoclones positive for one and not the other primer combination were crossed on a PDA plate, and the resulting mycelium was confirmed to form clamp connections using a light microscope. Protoclone strains were named according to their sampling origin at the fairy ring, isolate number, and nucleotype (arbitrarily assigned as A or B).

### DNA Extraction and Genome Sequencing.

The 12 mono- and dikaryotic strains (Fig. 2 and *SI Appendix*, Table S1) were grown in 2% liquid malt extract for 10 to 14 d on a rotary shaker. For Illumina sequencing, DNA was obtained from resulting mycelia and their source fruiting bodies using the Zymo Fungal/Bacterial miniprep kit. Sequencing libraries were prepared from 1 μg DNA using the TruSeq PCRfree DNA sample preparation kit (cat#20015962/3, Illumina) and unique dual indexes (cat#20022370, Illumina), targeting an insert size of 350 bp. The library preparation was performed according to the manufacturer’s instructions (guide #1000000039279). Sequencing was done using a NovaSeq 6000 SP flowcell, with paired-end 150-bp read length and v1 sequencing chemistry.

For Nanopore, we extracted and sequenced DNA only from the cultured strains. We used the Qiagen Genomic Tip 100/G following the manufacturer’s instructions, and isolated DNA was size selected for 25 kb or longer fragments using the Circulomics Short Read Eliminator Kit. No shearing was performed. Sequencing was done using two flowcells of the Nanopore PromethION system, base calling with ont-guppy-for-minknow 3.2.10, and reads were separated based on barcode using NanoComp.

### Short-Read Variant Calling and Testing for Mitotic Recombination.

The Illumina data were first investigated for ploidy using KAT (74), where the haploid monokaryotic condition was confirmed for protoclones and diploid/dikaryotic for dikaryons. Adapters were trimmed from the raw reads with Trimmomatic (75). From the eight protoclones, reads were mapped using BWA mem (76) to the Maror2 reference genome [(49); National Center for Biotechynology (NCBI) accession no. JAFLEJ000000000] and variants were called with the Genome Analysis Tool Kit (GATK), specifying a haploid ploidy level (77). Variants in repetitive regions and with more than two alleles were filtered out from the resulting file using VCFtools (78). Relationships of the protoclones were investigated by constructing a Neighbor Network with SplitsTree v4 (79).

To find mitotic recombination, we reasoned that a signal of recombination would be several variant sites in succession that had swapped genotype from one group of protoclones to the other. With this signal in mind, we started by separating the variant call set for the two nucleotypes A and B. We then searched the variant call subsets for sites with at least two variants in succession where the genotype diverged in a single protoclone. A few such sites were identified, but all of them were short, and after visual examination in IGV, they were discarded due to poor mapping quality of reads in these specific regions.

### De Novo Genome Assembly.

Adapters were trimmed from the raw Nanopore reads using Porechop (80). We created de novo assemblies for the eight monokaryotic strains (protoclones) the following way. We used miniasm (81) to get raw assemblies. Synteny of the raw assemblies to the *M. oreades* reference genome Maror2 was investigated using nucmer within MUMmer (82). Here we noticed a potential chimeric contig in E2B, which was confirmed as a misassembly by mapping back the raw reads with minimap2 (81) followed by inspection in IGV (83). This misassembly was manually split within Geneious (https://www.geneious.com/). After this correction, the assemblies were polished using one round of Racon (84) and one round of HyPo (85). After polishing, the assemblies were subjected to reference-guided scaffolding by first aligning the assemblies to the reference genome Maror2 with nucmer. We found that most chromosome sequences had been assembled to one or two contigs depending on the strain, and we merged these contigs using a custom script (https://github.com/markhilt/genome_analysis_tools/blob/master/overlap_scaffolding.py), that looks for alignments between contig ends before merging them, resulting where possible in gapless contigs, otherwise inserting a gap in the form of 100*N. Finally, to correct base errors in newly merged regions, we did one additional round of polishing with HyPo.

Combined assemblies for the two nuclear genotypes of the fairy ring were generated as follows. The four protoclones sharing the genotype (A or B) were treated as one sample for creating the combined assembly. Nanopore reads over 60 kb in length were extracted from read sets from each strain and assembled using miniasm. The two resulting combined assemblies were scaffolded similarly as the protoclone-specific assemblies. One gap remained in each assembly after scaffolding, and these were corrected manually within Geneious by trimming and overlapping the contig ends. Noting that some telomeres were missing, we created additional assemblies using Canu v2 (86) with all reads from all strains of the two nuclear genotypes, which successfully reconstructed most telomeres. We attached the resulting telomeres manually to the miniasm assemblies within Geneious. Finally, both assemblies were polished using the Nanopore reads over 60 kb and all Illumina reads from the protoclones of the two nuclear genotypes using HyPo.

### Comparative Genomics.

We used nucmer with parameters -b 2000 -c 2000 –maxmatch to align the combined assemblies of the two nuclei to each other. To annotate their repetitive content, we applied RepeatMasker v4.0.7 (www.repeatmasker.org/) with the Maror2 repeat library (49). The output was processed to calculate distributions along chromosomes on windows of 50 kb and steps of 10 kb using the utilities makewindows and coverage of BEDTools v2.29.0 (87, 88). The chromosome alignments and TE distributions were then plotted with Circos v0.69.6 (89). To investigate if the distribution of TE proportion per chromosome was different between the genotypes, we used the Kolmogorov–Smirnov two-sample test in R v3.5.1 (90) separately for the LTR and DNA element proportions.

### SV Analysis.

To find structural variants in the protoclones, we aligned each protoclone-specific genome assembly to the combined assembly of its nuclear genotype using minimap2. Variants were called using SyRI (91). Protoclones were compared to the other members of its genotype, and variants shared between all protoclones were filtered out, since they likely originated from misassemblies in the combined assembly. We considered only variants larger than 50 bp. Read mappings were visualized across all remaining variants using IGV (83).

We used a BLAST-based pipeline called “SpecificTE” inspired by Guichard et al. (92) to complement the structural variant detection above (available at https://github.com/SLAment/SpecificTE_Marasmius). In essence, we made pairwise comparisons between the protoclone assemblies of the same genotype, in search of sites that had a TE insertion in one protoclone (a “filled” site) that was absent in the other protoclone (an “empty” site). First, for every assembly, we extracted 5,000 bp flanking the 5′ and 3′ ends of every TE annotated with RepeatMasker larger than 120 bp. These flanks were used as query in BLAST searches against the other protoclone genomes. If the hits of both flanks were found to be within 20 bp of each other, in the same orientation, and with alignment length of at least 4,500 bp each, the locus was considered to be a putative empty site. We then created an artificial sequence by merging the flanks of the putative empty sites, and used it as query in BLAST searches against the assemblies to confirm its existence. Only putative empty sites with a single BLAST hit, a minimum 99% identity value, and at least 9,000 bp-long were considered further. To find homologous loci among the pair-wise putative empty sites, we made BLAST searches of the flanks surrounding the focal TE of all samples against themselves. For these putative homologous loci with TE insertions, we assumed that the flanking sequences cannot be farther apart from each other than 70 kb (that is, a TE insertion cannot be longer than 70,000 bp total – 9,000 flanks = 61,000 bp), based on the maximum size of the elements in our TE library (∼42 kb) and allowing for extra sequence. The sequences of each locus were then aligned with MAFFT v7.407 (93) with the option –adjustdirection and visually inspected to confirm the presence of a target site duplication when possible.

Noting that many SVs were present in the mosaic state, we suspected that more such variants could be present but not assembled, and thus impossible to find with the above two methods. Accordingly, we used Sniffles v1.0.12 (94) to look for SVs directly in the Nanopore reads mapped to their combined assembly by Minimap2 (81). Insertions and deletions larger than 50 bp were considered, and read mappings were visualized using IGV to confirm the variant calls (83).

### Phylogenetic Analysis of MarorhAT-10 and Associated Nonautonomous Elements.

By examining the structural variants detected above, we found a few insertions with a transposase-like protein with no known function (DUF659) and a *hAT* family C-terminal dimerization region. We used these insertions to define a consensus of the full autonomous element MarorhAT-10, as described in Hiltunen et al. (49). We then used this MarorhAT-10 consensus to extract all similar sequences in the protoclone assemblies based on BLAST searches with the script “query2haplotype.py” v1.41 (https://github.com/SLAment/Genomics/blob/master/BLAST/query2haplotype.py) and parameters –minhaplo 300 –vicinity 3600. In the case of tandem insertions, only one sequence was considered. We also compared the full element with all elements present in the Maror2 TE library using BLASTn, and retained all hits with an e-value smaller than 0.0001. The TIR sequences were determined by aligning each element to its own reverse complement. All surviving sequences were aligned with MAFFT and manually curated to retain only the hits that had both TIRs and no nested insertion of another TE. The cleaned alignment was given to IQ-TREE v1.6.8 (95, 96) to infer a maximum-likelihood tree with extended model selection (–m MFP) and 100 standard bootstrap pseudoreplicates.

### TE Movement in Single-Spore Isolates.

We investigated TE activity in monokaryons derived from single sexual basidiospores. For this purpose, we utilized our previously published dataset of 95 single-spore isolates (49), originating from four fruiting bodies from the same fairy ring genet as in the present study, but from another year [2017 in the present study, 2018 in Hiltunen et al. (49)]. In short, spore prints were obtained from the fruiting bodies by removing the stipe and placing the cap onto a Petri dish with the hymenium facing down overnight. The next day, the cap was removed and the spore print dissolved in sterile H_2_O. Dilution series were prepared and spread onto culture plates containing PDA. Plates were monitored for up to a week after inoculation, and newly germinated spores were transferred to new PDA plates. DNA was extracted and sequencing libraries were prepared for each isolate separately, and resulting libraries were sequenced using the Illumina HiSeq X system to approximately 20× depth of coverage per genome.

We used the McClintock pipeline (97) to probe for novel TE insertions in the single-basidiospore dataset. This pipeline incorporates a multitude of different TE calling tools, and we included all of them in our analysis. The Maror2 genome (49) was used as a reference. In addition to the 95 single-spore isolates, we used the same pipeline to find TE insertions in the four dikaryotic strains that were subjected to protoplasting, the four fruiting bodies we obtained these dikaryons from, in addition to the four fruiting bodies from where the single-spore dataset was obtained, and dikaryotic strains from three of these fruiting bodies (one of them ceased to grow before DNA extraction). Reasoning that true positive new insertions likely could be found only in single samples, we removed any calls that overlapped in the genome with calls in any other sample, including both mono- and dikaryons. Only nonreference calls were considered. In addition, to filter out false-positive calls, we considered only TEs identified by at least three of the TE identification modules of McClintock. We additionally ran the pipeline across the dikaryotic Illumina data, (i.e., originating from fruiting bodies and dikaryotic cultures) and found no new TE insertions in those data.

### Methylation Analysis.

The mCpG frequency was quantified using Nanopolish v0.13.2 (54). Raw fast5 files from all 12 strains where we obtained Nanopore sequencing data were indexed using Nanopolish index. Next, we mapped reads to the combined assemblies using Minimap2 (81): all nucleotype A protoclones to the A combined assembly and all B protoclones to the B assembly. All dikaryons were mapped to both combined assemblies. To exclude reads from the other nucleotype in the dikaryon data (i.e., reads from B when mapping to the A assembly), we required mapped reads to: 1) have a mapq value of 60, 2) be primary alignments, and 3) have an uninterrupted mapping distance of at least 6 kb. Samtools v1.7 and samjs v2927d9787 (98) were used to filter bam files. For consistency, the same filters were applied to bam files from the protoclones. Nanopolish call-methylation and calculate_methylation_frequency.py were used to calculate the frequency of 5-methylcytosine in reads mapping at each CpG site across the genome. Genomic regions with active TEs were extracted using BEDtools (87). For the comparison of repeats versus nonrepeat regions, the number of sites was downsampled 500 times prior to statistical analysis, which was performed in R v4.1.2 (90).

### Population Coefficient of Variation in *M. oreades* TE Families.

To estimate variation in TE abundance in the *M. oreades* population, we used our previously generated Illumina reads originating from six different fairy rings (8) in addition to our newly generated data for ring 43 [samples 05E, 01S, 03W, 06E2, 04SE2, 08N from the previous study (8), N2017fb from the present, all originating from dikaryotic fruiting body stipe tissue]. Median coverage was calculated for each read set by mapping to the *M. oreades* reference genome and focusing only on single copy ortholog regions as identified by BUSCO v5.2.2 with the Agaricomycetes_odb10 database (99). Read sets were normalized to 35× per sample by random subsampling of reads with seqtk v1.3-r106 (https://github.com/lh3/seqtk), and mapped to the *M. oreades* repeat library (49) that had been supplemented by the newly identified TE families in this study. Median depth of coverage for each TE family was used as an estimate of its abundance in the source genome. To avoid issues of partial mapping (e.g., because of solo LTRs), we required horizontal coverage (minimum 15×) across 90% of each TE, otherwise we considered the TE to be absent in the genome and coded the median as 0. The coefficient of variation (SD/mean) in median coverage across all samples was calculated for each TE family in R v4.1.2 (90).

## Supplementary Material

Supplementary File

Supplementary File

## Data Availability

All data generated during this study, including raw Nanopore and Illumina sequencing reads and de novo assemblies of the combined nucleotypes A and B, are available in the NCBI BioProject PRJNA838774 (100) (Sequence Read Archive accessions: SRR19239449–SRR19239476 (101, 102); Genome accessions: CP097446–CP097456 (103, 104) for nucleotype A, CP097435–CP097445 (105, 106) for nucleotype B; BioSamples for the protoclone strains have the “pc” addition to distinguish them from previous entries). The *M. oreades* repeat library hosted at Dryad has been updated with the newly discovered transposon families (https://datadryad.org/stash/dataset/doi:10.5061/dryad.000000034) (49).
