# Supplementary material for: Stage-specific transposon activity in the life cycle of the fairy-ring mushroom Marasmius oreades
Source: Proc Natl Acad Sci U S A. 2022 Nov 7;119(46):e2208575119. doi: 10.1073/pnas.2208575119 (PMC9674265; doi:10.1073/pnas.2208575119)
Supplement: Supplementary File [file pnas.2208575119.sapp.pdf]

PNAS

Supplementary Information for
Stage-specific transposon activity in the life cycle of the fairy-ring mushroom *Marasmius oreades*

Markus Hiltunen[1]*, Sandra Lorena Ament-Velásquez[1,2], Martin Ryberg[1], Hanna Johannesson[1,3]

[1]Department of Organismal Biology, Uppsala University, SE-752 36 Uppsala, Sweden
[2]Department of Zoology, Stockholm University, SE-106 91 Stockholm, Sweden (current affiliation)
[3]The Royal Swedish Academy of Sciences and Department of Ecology, Environment and Plant Sciences, Stockholm University, SE-106 91 Stockholm, Sweden (current affiliation)

*Corresponding author: Markus Hiltunen, Norbyvägen 18D, 75236, Evolutionary Biology Center, Uppsala, Sweden
Email: markus.hiltunen@ebc.uu.se

This PDF file includes:

Figures S1 to S5
Tables S1 to S2
Dataset S1

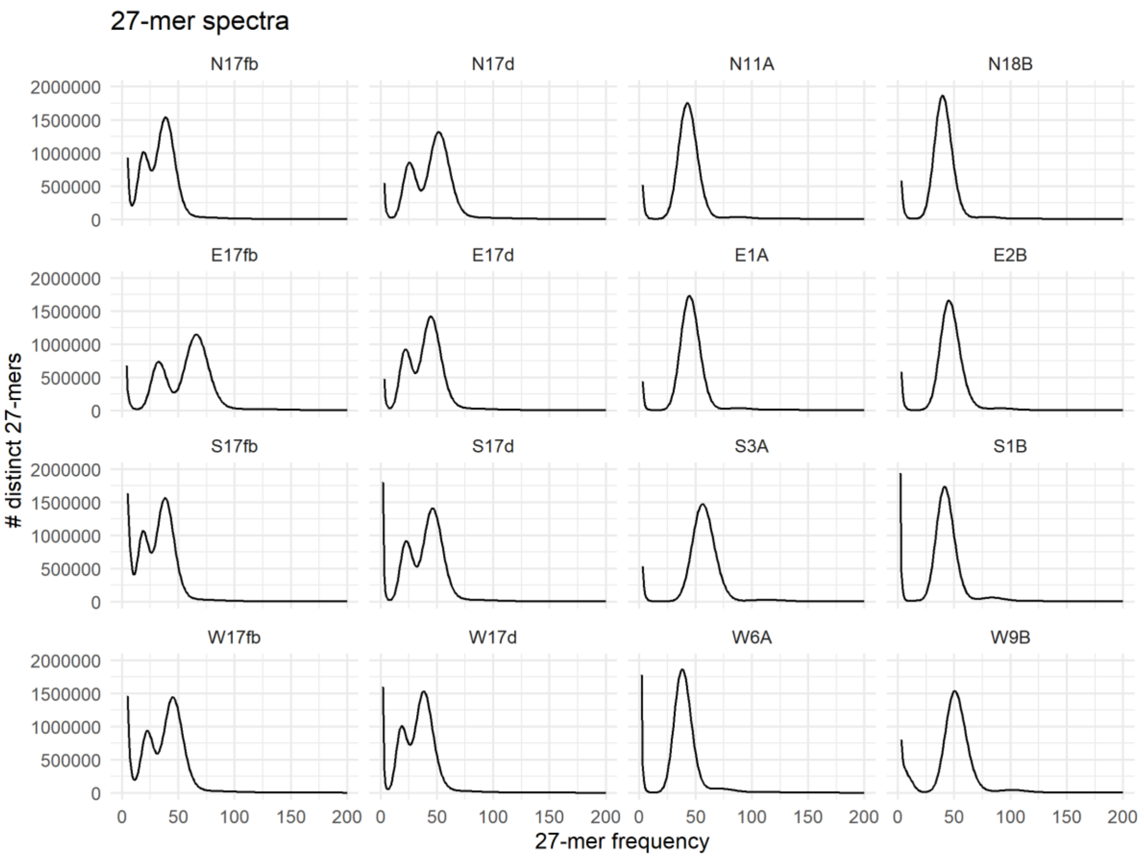

Fig. S1. 27-mer spectra of the raw Illumina data for the 16 samples (Figure 2; Supplementary Table S1). A single peak is consistent with the data containing a single genotype, i.e. haploidy and monokaryosis; a second peak indicates the presence of a variant genotype, such as during diploidy or dikaryosis.

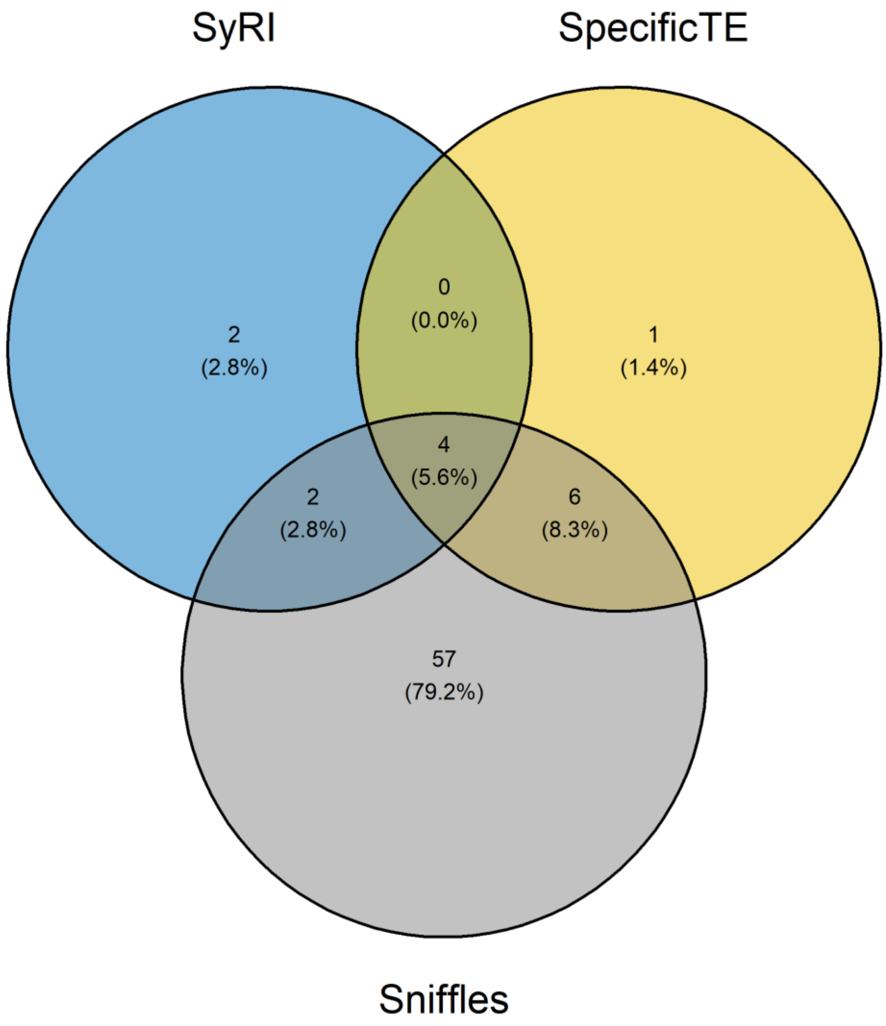

Fig. S2. A combination of SV callers was used to maximize variant discovery. In the Venn diagram, numbers indicate how many variants were discovered by each pipeline, with overlaps between methods also shown.

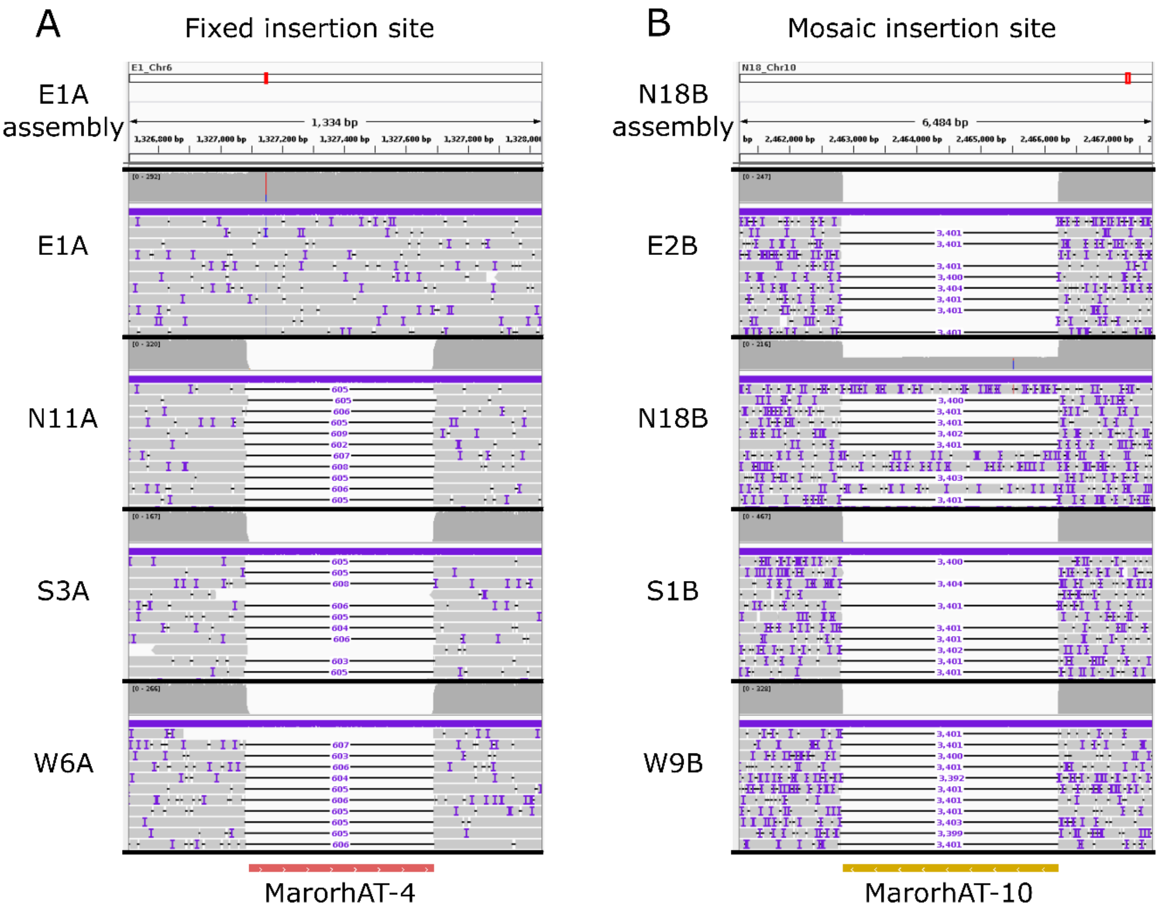

Fig. S3. Examples of fixed and mosaic transposon insertions shown in IGV. A. An insertion of MarorhAT-4 in the genome of E1A, present in all reads from this protoclone (fixed). B. An insertion of MarorhAT-10 in N18B, present in some of the reads (mosaic). In both A and B, the upper track corresponds to depth of coverage, while the lower track shows examples of mapped reads to the locus. Only reads with unique mapping are shown.

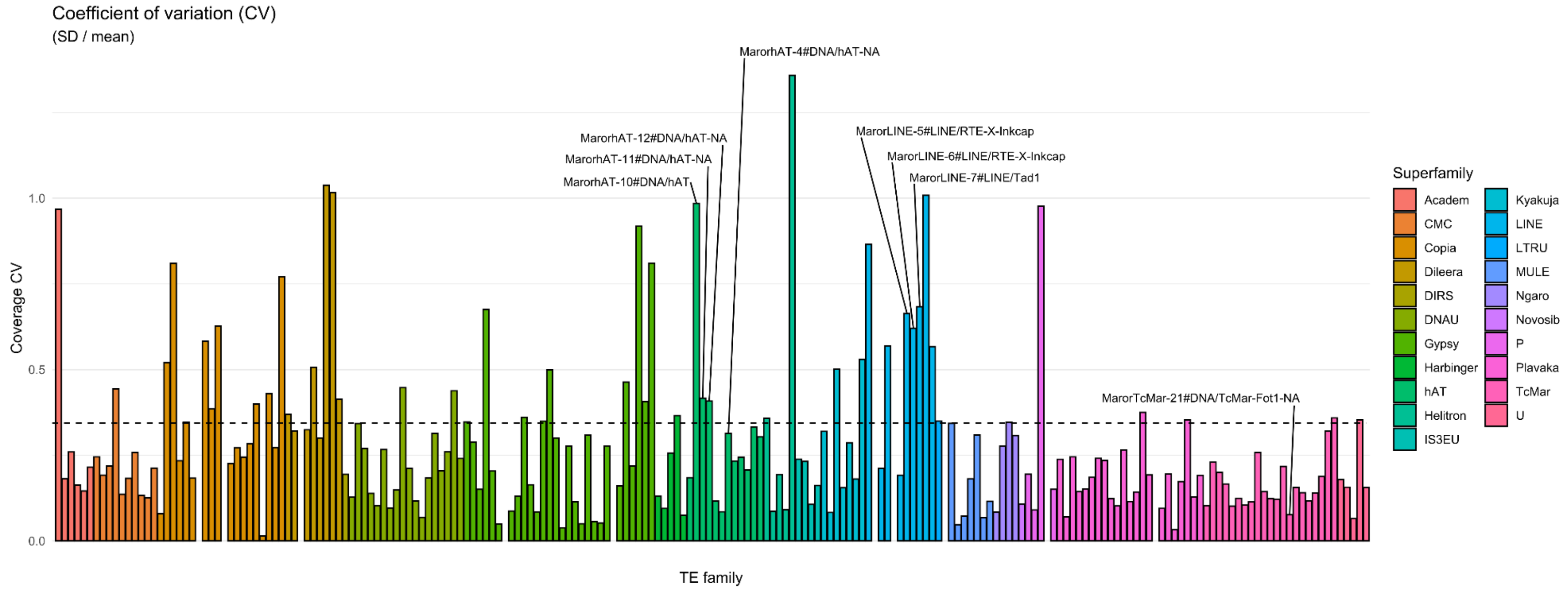

Fig. S4. Coefficient of variation (CV) in *Marasmius oreades* transposable element (TE) families. The CV was calculated based on normalized depth of sequencing coverage from different *M. oreades* individuals. A high CV indicates a high variance in TE load between the genomes of the seven individuals. Each bar corresponds to a TE family and superfamilies are color coded. Families found to be actively moving in the present study are indicated with their names above the bar. The dashed line indicates the third quartile.

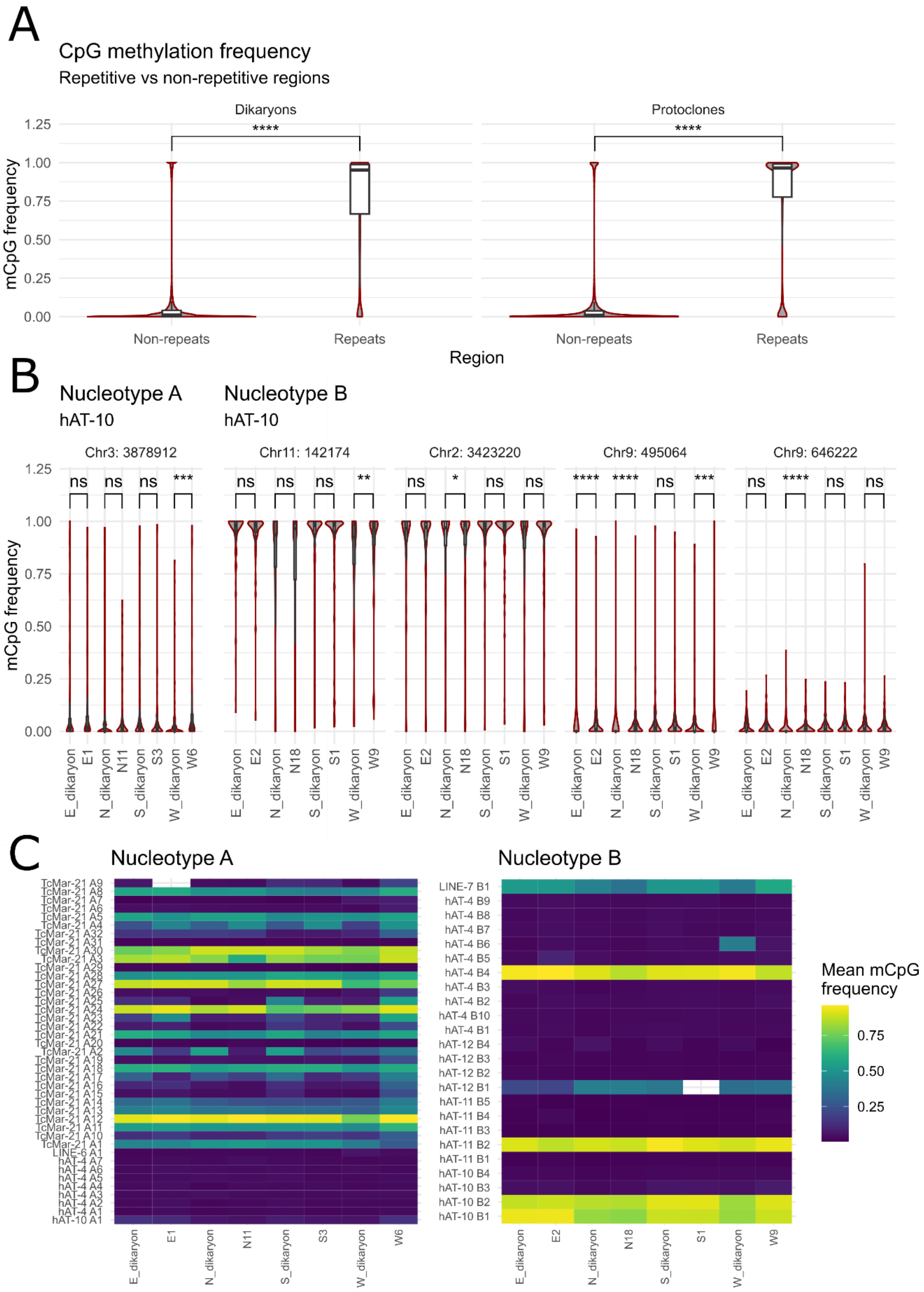
A
CpG methylation frequency
Repetitive vs non-repetitive regions
Dikaryons
Protoclones
****
****
mCpG frequency
1.25
1.00
0.75
0.50
0.25
0.00
Non-repeats
Repeats
Region
B
Nucleotype A
hAT-10
Nucleotype B
hAT-10
Chr3: 3878912
Chr11: 142174
Chr2: 3423220
Chr9: 495064
Chr9: 646222
ns
*
**
***
****
E_dikaryon
E1
E2
N_dikaryon
N11
N18
S_dikaryon
S3
S1
W_dikaryon
W6
W9
C
Nucleotype A
TcMar-21 A9
TcMar-21 A8
TcMar-21 A7
TcMar-21 A6
TcMar-21 A5
TcMar-21 A4
TcMar-21 A32
TcMar-21 A31
TcMar-21 A30
TcMar-21 A3
TcMar-21 A29
TcMar-21 A28
TcMar-21 A27
TcMar-21 A26
TcMar-21 A25
TcMar-21 A24
TcMar-21 A23
TcMar-21 A22
TcMar-21 A21
TcMar-21 A20
TcMar-21 A2
TcMar-21 A19
TcMar-21 A18
TcMar-21 A17
TcMar-21 A16
TcMar-21 A15
TcMar-21 A14
TcMar-21 A13
TcMar-21 A12
TcMar-21 A11
TcMar-21 A10
TcMar-21 A1
LINE-6 A1
hAT-4 A7
hAT-4 A6
hAT-4 A5
hAT-4 A4
hAT-4 A3
hAT-4 A2
hAT-4 A1
hAT-10 A1
Nucleotype B
LINE-7 B1
hAT-4 B9
hAT-4 B8
hAT-4 B7
hAT-4 B6
hAT-4 B5
hAT-4 B4
hAT-4 B3
hAT-4 B2
hAT-4 B10
hAT-4 B1
hAT-12 B4
hAT-12 B3
hAT-12 B2
hAT-12 B1
hAT-11 B5
hAT-11 B4
hAT-11 B3
hAT-11 B2
hAT-11 B1
hAT-10 B4
hAT-10 B3
hAT-10 B2
hAT-10 B1
Mean mCpG frequency
0.75
0.50
0.25

Fig. S5. Frequency of cytosine methylation at CpG sites (mCpG) in dikaryons and monokaryotic protoclones. A-B. Violin plots overlaid with boxplots (thick lines in the boxplots correspond to median values). Each datapoint corresponds to the methylation frequency in Nanopore reads at a single CpG site. A. Genomewide repeat regions compared to the rest of the genome. The number of sites was downsampled 500 times. B. Frequency of mCpG at each of the MarorhAT-10 source copies, one in nucleotype A and four in B. In all panels, significance levels of the Wilcoxon rank-sum test are indicated above each comparison (ns: $p > 0.05$; *: $p <= 0.05$; **: $p <= 0.01$; ***: $p <= 0.001$; ****: $p <= 0.0001$). C. Mean methylation frequency per copy for each active TE family in the two nucleotypes. Missing data shown as empty elements in the matrix. Numbering of element copies is independent between nucleotypes A and B and the same number does not necessarily indicate homology. Families inferred to be autonomous are hAT-10 and LINE-6, the rest are putatively non-autonomous families.

Table S1. Samples collected and data generated for the study.

Sample ID	Lab ID	Tissue type	Nucleotype	Nanopore N50 read length	Nanopore mean depth of coverage (X)[1]	Illumina mean depth of coverage (X)[1]
E17fb	E fruiting body	Fruiting body stipe	A+B	NA	NA	164
N17fb	N fruiting body	Fruiting body stipe	A+B	NA	NA	96
S17fb	S fruiting body	Fruiting body stipe	A+B	NA	NA	96
W17fb	W fruiting body	Fruiting body stipe	A+B	NA	NA	113
E17d	E dikaryon	Dikaryotic mycelium	A+B	21306	143	112
N17d	N dikaryon	Dikaryotic mycelium	A+B	23068	95	129
S17d	S dikaryon	Dikaryotic mycelium	A+B	26936	418	116
W17d	W dikaryon	Dikaryotic mycelium	A+B	27692	186	97
E1A	E1	Monokaryotic mycelium (protoclone)	A	32733	281	114
N11A	N11	Monokaryotic mycelium (protoclone)	A	32805	296	108
S3A	S3	Monokaryotic mycelium (protoclone)	A	33162	164	144
W6A	W6	Monokaryotic mycelium (protoclone)	A	30038	241	98
E2B	E2	Monokaryotic mycelium (protoclone)	B	31935	266	111
N18B	N18	Monokaryotic mycelium (protoclone)	B	24932	248	98
S1B	S1	Monokaryotic mycelium (protoclone)	B	26940	429	103
W9B	W9	Monokaryotic mycelium (protoclone)	B	27719	367	124

[1] Calculated from a subset of the reference genome.

Table S2. Genome assemblies generated during the study.

Assembly	Nucleotype	N Gaps	N Telomeres	Genome proportion of combined assembly (%)	Busco report[1]
A combined	A	0	19	NA	C:96.3%[S:95.6%,D:0.7%],F:2.2%,M:1.5%,n:1335
B combined	B	0	18	NA	C:96.1%[S:95.5%,D:0.6%],F:2.2%,M:1.7%,n:1335
E1A	A	1	11	96.25	C:95.9%[S:95.2%,D:0.7%],F:2.3%,M:1.8%,n:1335
S3A	A	0	15	96.40	C:96.1%[S:95.4%,D:0.7%],F:2.3%,M:1.6%,n:1335
W6A	A	0	13	96.42	C:96.2%[S:95.5%,D:0.7%],F:2.2%,M:1.6%,n:1335
N11A	A	0	13	96.51	C:96.2%[S:95.5%,D:0.7%],F:2.3%,M:1.5%,n:1335
E2B	B	0	13	96.19	C:96.2%[S:95.6%,D:0.6%],F:2.2%,M:1.6%,n:1335
S1B	B	2	11	96.19	C:95.8%[S:95.1%,D:0.7%],F:2.4%,M:1.8%,n:1335
W9B	B	3	13	96.17	C:96.1%[S:95.4%,D:0.7%],F:2.1%,M:1.8%,n:1335
N18B	B	1	10	96.21	C:95.8%[S:95.2%,D:0.6%],F:2.3%,M:1.9%,n:1335

[1] C: Complete, S: Complete and single-copy, D: Complete and duplicated, F: Fragmented, M:Missing, n: Number of Busco groups searched

Dataset S1 (separate file). Identified structural variants.
